# Active Targeting Strategies Using Biological Ligands for Nanoparticle Drug Delivery Systems

**DOI:** 10.3390/cancers11050640

**Published:** 2019-05-08

**Authors:** Jihye Yoo, Changhee Park, Gawon Yi, Donghyun Lee, Heebeom Koo

**Affiliations:** 1 Department of Medical Life Sciences, College of Medicine, The Catholic University of Korea, 222 Banpo-daero, Seocho-gu, Seoul 06591, Korea; yooji0498@catholic.ac.kr (J.Y.); parkch@catholic.ac.kr (C.P.); gawon4292@catholic.ac.kr (G.Y.); a2168989@catholic.ac.kr (D.L.); 2 Department of Biomedicine & Health Sciences, College of Medicine, The Catholic University of Korea, 222 Banpo-daero, Seocho-gu, Seoul 06591, Korea; 3 Catholic Photomedicine Research Institute, College of Medicine, The Catholic University of Korea, 222 Banpo-daero, Seocho-gu, Seoul 06591, Korea

**Keywords:** nanoparticle, drug delivery, ligand, active targeting, tumor targeting, biodistribution

## Abstract

Targeting nanoparticle (NP) carriers to sites of disease is critical for their successful use as drug delivery systems. Along with optimization of physicochemical properties, researchers have focused on surface modification of NPs with biological ligands. Such ligands can bind specific receptors on the surface of target cells. Furthermore, biological ligands can facilitate uptake of modified NPs, which is referred to as ‘active targeting’ of NPs. In this review, we discuss recent applications of biological ligands including proteins, polysaccharides, aptamers, peptides, and small molecules for NP-mediated drug delivery. We prioritized studies that have demonstrated targeting in animals over in vitro studies. We expect that this review will assist biomedical researchers working with NPs for drug delivery and imaging.

## 1. Introduction

The site where a drug is administered is most often very distant from the site of expected therapeutic effect. Thus, there has been significant research in the field of targeted drug delivery. Nanoparticle (NP) drug delivery systems represent the cumulative research efforts of numerous fields, including chemistry, biology, material science, pharmaceutics, and clinical medicine [1]. Commercialized formulations such as Doxil and Abraxane are excellent examples of NP drug delivery systems that have improved therapy in patients [2,3]. NPs can disperse hydrophobic drugs stably in aqueous conditions without aggregation. [4]. Importantly, their physicochemical properties, including size and surface charge, can easily be modified by adjusting the component molecules or fabrication method [5]. NPs can delay early release of drugs in order to allow sufficient time for therapeutic action. NPs also allow for controlled release of drugs, which in some cases can be tailored to respond to specific stimuli such as pH, light, heat, or enzymes [6].

With respect to targeted drug delivery, NPs utilize two basic strategies comprising either passive or active targeting [7]. Passive targeting is based on physicochemical properties [8]. Specifically, when NPs employing a passive targeting release method are injected intravenously, they generally circulate longer in the blood stream compared to free drugs. In angiogenic tissues such as tumors, NPs employing passive targeting penetrate the fenestrated structure of blood vessels more at the disease site, which in turn leads to significant accumulation of the drug, which is aided in part by slow lymphatic drainage. This scenario is referred to as the enhanced permeability and retention (EPR) effect. The EPR effect is supported by promising data from many reports on NPs [9]. Compared to passive targeting, active targeting relies on a biological interaction between ligands on the surface of NPs and the cell target.

**Scheme 1:**
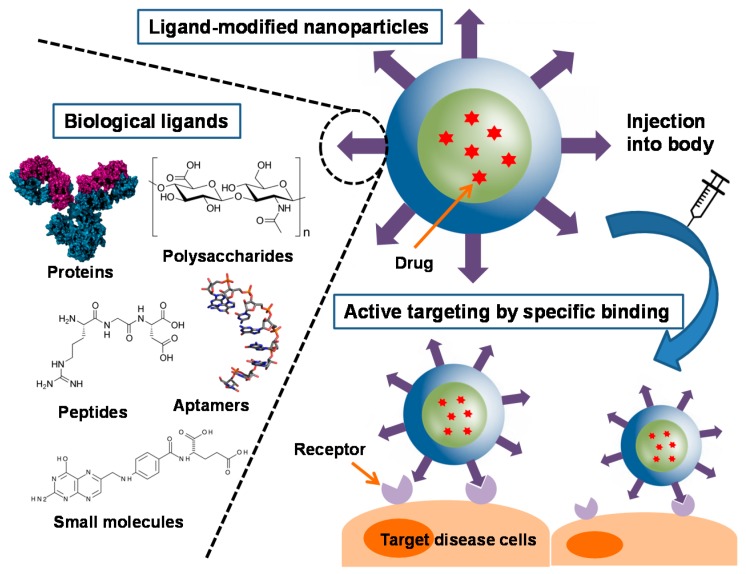
Illustration of biological ligands for active targeting of nanoparticle drug carriers.

A large number of biological ligands have been identified and studied for facilitating active targeting of NPs [10]. Such biological ligands often bind to specific receptors on the surface of the target cells, and in this way increase cellular uptake of drug-containing NPs and also increase therapeutic efficacy [11]. Compared to singular ligand, an increased density of ligands is advantageous for promoting binding and cellular uptake through the multivalent effect [12]. Various types of ligands have been employed for this purpose, including proteins, polysaccharides, nucleic acids, peptides, and small molecules (Scheme 1). Generally, NPs are functionalized with these ligands by two ways. They can be chemically conjugated or physically adsorbed on the NPs after formation of NPs, or can be linked with NP components, such as polymers, before formation [13,14]. In this review, we discuss different types of biological ligands and review their current applications in NP-based drug delivery systems, focusing primarily on studies reporting promising outcomes in vivo (Table 1).

## 2. Biological Ligands and Their Applications for Nanoparticles

### 2.1. Proteins and Polysaccharides

**Figure 1:**
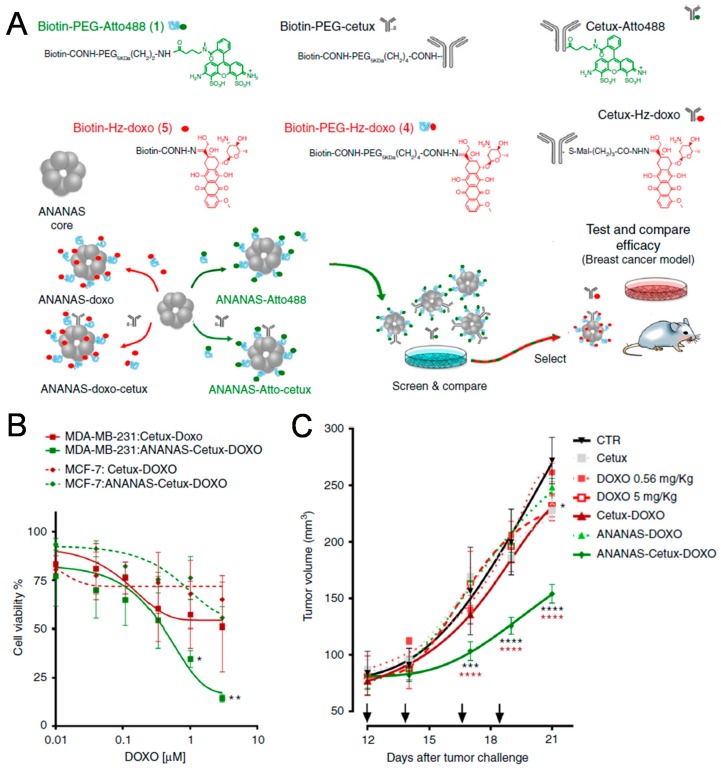
Antibody-conjugated nanoparticles (NPs). (**A**) Schematic illustration of antibody-guided avidin-nucleic-acid nanoassemblies (ANANAS) modified with antiepidermal growth factor receptor (EGFR) antibody (cetuximab) for enhancing cancer targeting. (**B**) Cell viability assay of cetuximab-guided ANANAS in MCF-7 and MDA-MB-231 cell lines. (**C**) Tumor growth in mice bearing MDA-MB-231 tumors after intravenous injection of cetuximab-guided ANANAS. Reproduced with permission from Reference [18].

Among biological ligands, antibodies have the longest history with respect to targeting specific receptors [15]. Antibodies are tens of kilodaltons in size and have high specificity, consistent with the generalized trend that larger ligands exhibit more specific binding [16]. In particular, many antibodies can be used not only for targeting, but for therapeutic purposes as well [17]. Nevertheless, the large size of antibodies limits their density on the surface of NPs during modification. In 2018, Roncato et al. reported the use of antiepidermal growth factor receptor (EGFR) antibody (cetuximab) in antibody-guided avidin-nucleic-acid nanoassemblies (ANANAS) for efficient cancer therapy (Figure 1) [18]. Specifically, they synthesized poly-avidin cores combined with biotin-conjugated molecules. They evaluated the targeting efficacy of the antibody–drug conjugates (ADCs), which are widely used in personalized cancer therapy. The authors found that cetuximab-guided ANANAS could increase the drug–antibody ratio more than ADCs, which they attributed to the ability of the avidin–biotin interaction to increase the drug capacity of ANANAS carriers. In that same study, ANANAS were modified with biotin-poly (ethylene glycol) (PEG)-cetuximab for targeting, biotin-PEG-Atto488 for imaging, and biotin-hydrazine-doxorubicin for therapy. They also investigated hydrazine bonds, which are acid-reversible and can release drugs under mild acidic environments such as lysosomes after cellular uptake. Cetuximab-guided ANANAS exhibited faster cellular internalization than both untargeted ANANAS and antibody alone in MDA-MB-231 (EGFR-expressing cells). In MDA-MB-231 tumor-bearing mice, cetuximab-guided ANANAS-treated groups showed improved therapeutic efficacy compared to other groups because of their high drug–antibody ratio and targeting ability with cetuximab.

Affibodies (Afbs) are engineered, high-affinity proteins that are smaller than normal antibodies [19]. Recently, Oh et al. successfully used Afbs for active targeting of NPs [20]. To avoid clearance of NPs by the mononuclear phagocyte system (MPS), Oh et al. suggested a protein corona shield concept. They decorated the surface of mesoporous silica NPs (MSNs) containing camptothecin (CPT) with human epidermal growth factor receptor 2 (HER2) binding Afbs by supramolecular interaction. The resulting Afb-CPT-MSNs were approximately 270 ± 20 nm in size and had superior colloidal stability that afforded extended blood circulation time without the need for a traditional polyethyleneglycol (PEG) coating. They showed fast cellular uptake in the HER2-receptor-overexpressing SK-BR-3 breast cancer cell line, but not in control MCF-10A cells. Interestingly, in RAW264.7 murine macrophage-like cells, Afb-CPT-MSNs exhibited reduced internalization compared to free CPT, which was otherwise highly cytotoxic. In SK-BR-3 tumor-bearing mice, the Afb-modified NPs accumulated to higher levels in tumor tissue after intravenous injection compared to PEG-coated control NPs. Furthermore, they inhibited tumor growth by approximately 90%, and were found to escape from reticuloendothelial organs during ex vivo experiments. Taken together, these results demonstrated that Afb modification of NPs by protein engineering could be used to enhance the stealth effect of NPs in order to facilitate better escape from MPS compared to PEG coating while minimizing serum protein adsorption due to the presence of a protein corona.

Transferrin (Tf) is an iron-binding glycoprotein that is responsible for iron transport in the body [21]. Tf receptors are highly expressed in specific tissues and cells, which can be targeted by Tf-modified NPs. The Davis group used conjugated NPs to target Tf receptors on the blood side of the blood–brain barrier (BBB) to deliver therapeutic drugs through the BBB [22]. They anticipated that Tf-modified NPs that specifically bind Tf receptors would be unable to pass through the BBB, and thus be limited to the blood side of the BBB. To overcome this potential limitation, they used the acid-cleavable linkage DAK [2,2-bis-(aminoethoxy)-propane] to conjugate Tf to NP cores. DAK exhibits good stability at a neutral pH, with a hydrolysis half-life of 60 min at pH 5.5. Owing to the nature of the DAK linkage, transcytosis of Tf-modified NPs results in separation from the NP core under acidic environments and subsequent release into the parenchyma for therapy. Importantly, the Davis group showed that the cleavable DAK moiety was helpful in facilitating specific delivery to the brain, with an ideal ratio of 200 Tf molecules per NP for this purpose. This strategy of using Tf-modified NPs and cleavable linkages supports the possibility of NP-mediated drug delivery in brain disease, and further drug applications are expected.

Hyaluronic acid (HA) is a polymer capable of binding cell surface receptors for active targeting. HA is a polysaccharide and one the main components of the extracellular matrix along with collagen. HA binds CD44, which is often overexpressed on the surface of cancer cells and is believed to be a representative marker of cancer stem cells [23]. Interestingly, HA can be used simultaneously as a hydrophilic backbone polymer of NPs and a targeting moiety as demonstrated by Choi et al. [24]. Furthermore, HA is degraded by the enzyme hyaluronidase 1 (Hyal-1), which is highly expressed in various malignant cells and thus can accelerate drug release in target tissue. In the study by Choi et al., the HA-NPs consisted primarily of HA modified with PEG and hydrophobic cholanic acids to create a self-assembled amphiphilic structure. The as-prepared HA-NPs were not sequestered by the reticuloendothelial system (RES) and exhibited long blood circulation times, which in turn promoted specific accumulation in tumors. In one study, hydrophobic camptothecin (CPT) was loaded into the HA-NPs as an anticancer therapeutic. In the presence of Hyal-1, the CPT-loaded HA-NPs were rapidly degraded, which in turn led to quick release of CPT. In cancer cells, the CPT-HA-NPs showed dose-dependent cytotoxicity, but their cytotoxicity was highly reduced in normal cells with low CD44 expression. After that, the tumor-targeting capability of CPT-HA-NPs as well as their antitumor effect were also demonstrated in SCC7 and MBA-MB-231 tumor-bearing mice models.

### 2.2. Peptides

Among targeting ligands, peptides have several advantages such as low cost of production, good stability, and ease of conjugation to the surface of NPs at a high density due to their small size [25]. To target interleukin-4 receptor (IL-4R) expressed in both lung tumor cells and tumor endothelial cells, Chi et al. reported using an IL-4R-binding peptide-1 (IL4RPep-1) with the sequence CRKRLDRNC identified using a phage-display technique [26]. The IL4RPep-1 showed excellent binding to H226 human lung cancer cells overexpressing IL-4R and was stable in whole serum for up to 4 h. Based on these findings, the authors further developed IL4RPep-1-labeled liposomes incorporating doxorubicin (IL4RPep-1-L-Dox). Cell binding and uptake of IL4RPep-1-L-Dox were more efficient than that of unlabeled liposomes (L-Dox) due to the peptide moiety. Intravenously injected IL4RPep-1-L-Dox into H226 tumor-bearing mice was also found to accumulate more significantly and had greater antitumor activity compared to L-Dox without peptide. Using immunofluorescence, the authors demonstrated that IL4RPep-1-L-Dox was present in vascular endothelial cells of tumor tissues. Taken together, these data confirmed successful targeting of IL4RPep-1-L-Dox to tumor blood vessels with concomitant improvement in chemotherapeutic efficacy.

**Figure 2:**
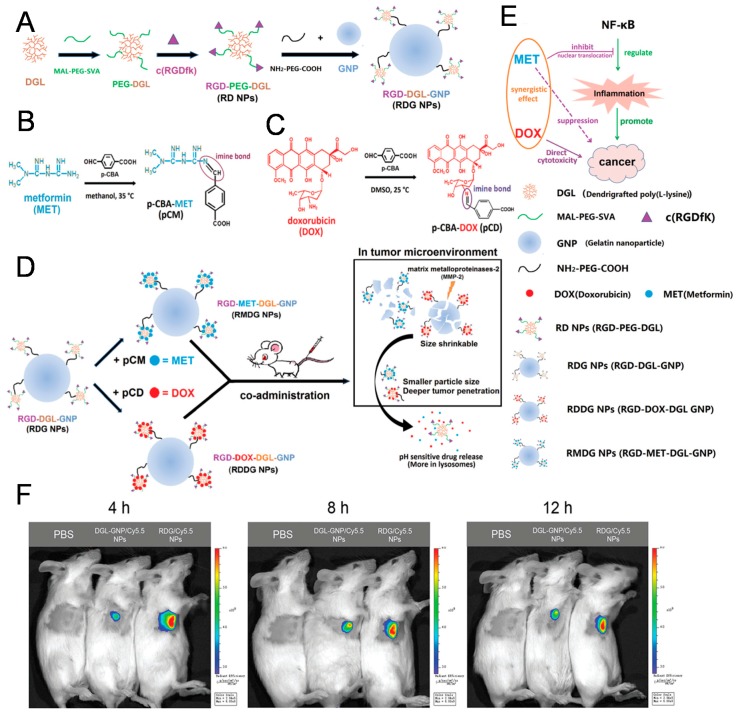
Peptide-conjugated NPs. Schematic illustration of RGD-doxorubicin (DOX)-dendri-grafted poly-L-lysine (DGL)-gelatin nanoparticles (GNP) (RDDG NPs) and RGD-metformin (MET)-DGL-GNP (RMDG NPs). (**A**) Synthetic procedure for RGD-DGL-GNP (RGD NPs). Chemical structure and synthetic procedure for (**B**) 4-Carboxybenzaldehyde (p-CBA)-MET (pCM) and (**C**) p-CBA-DOX (pCD). Illustration of the RDDG NPs and RMDG NPs in the tumor microenvironment (**D**) and their antitumor/anti-inflammatory effects (**E**). (**F**) In vivo fluorescence images showing tumor accumulation of NPs with or without RGD ligand. Reproduced with permission from Reference [29].

The arginylglycylaspartic acid (RGD) peptide binds integrins, which are particularly overexpressed in vascular endothelial cells present in tumor tissue, and for this reason is a well-known tumor-targeting peptide [27]. The sequence of the RGD peptide originates from cell attachment proteins including fibronectin, vitronectin, and laminin [28]. In 2018, Lu et al. developed size-shrinkable NPs for enhanced cancer therapy using the RGD peptide as the targeting ligand (Figure 2) [29]. For deeper penetration into tumor tissue, small NPs containing metformin (MET) or doxorubicin (DOX) were linked to the surface of large gelatin nanoparticles (GNP). In addition, Lu et al. generated small NPs containing RGD peptides as well as therapeutic agents to target tumors with overexpression of integrin. They used MET as an anti-inflammatory drug for combination with DOX, and both drugs were conjugated to small NPs via degradable imine bonds. Large GNPs can be degraded by matrix metalloproteinases-2 (MMP-2) overexpressed in cancer, while small NPs decorated with RGD peptide can easily penetrate deeply into tumor tissues. MET and DOX are then released from small NPs after degradation of imine bonds in the acidic environment of tumor tissues. In this way, nuclear factor-ĸB (NF-ĸB) inducing cancer-related inflammation can be inhibited by MET, while DOX exerts cytotoxic effects in cancer cells. Both GNP and RGD NPs exhibit improved accumulation in 4T1 and CT26 tumors via targeting ligand RGD and size shrinkage. Coadministration of MET and DOX-containing NPs revealed superior antitumor and antimetastatic effects in 4T1 and CT26 tumor-bearing mice. Furthermore, the anti-inflammatory effect of MET-containing NPs was successfully evaluated during analysis of TNF-α, NF-ĸB, IL-6, and Ki67.

In addition to the RGD peptide, the iRGD peptide represents an RGD-containing peptide sequence initially characterized by its ability to bind αv integrins expressed on tumor endothelial cells. Interestingly, when the iRGD peptide is cleaved by proteases in tumor cells, it produces a CRGDK/R derivative peptide that has diminished affinity for αv integrin but increased affinity for neuropilin-1 (NRP-1) [30]. This switch in affinities promotes tumor-specific penetration of molecules due to the presence of the CendR peptide motif. These unique characteristics of the iRGD peptide make it useful for enhanced drug delivery in tumor tissues. For example, the Ruoslahti group showed that the iRGD peptide produced increased tumor-specific vascular permeability in five tumor models compared to a control peptide lacking the CendR motif [31]. In particular, even when not conjugated to drugs or NPs, the iRGD peptide effectively increased particle tumor accumulation owing to the natural structure of the iRGD and CendR motifs. Therefore, iRGD combination groups with free drugs or NPs enhance cancer therapy compared to free drug or NP alone. This special ability of iRGD is different from other biological ligands and may be worth pursuing in the future as a way to reduce the amount of chemotherapy needed to treat certain types of cancer [32].

### 2.3. Aptamers

**Figure 3:**
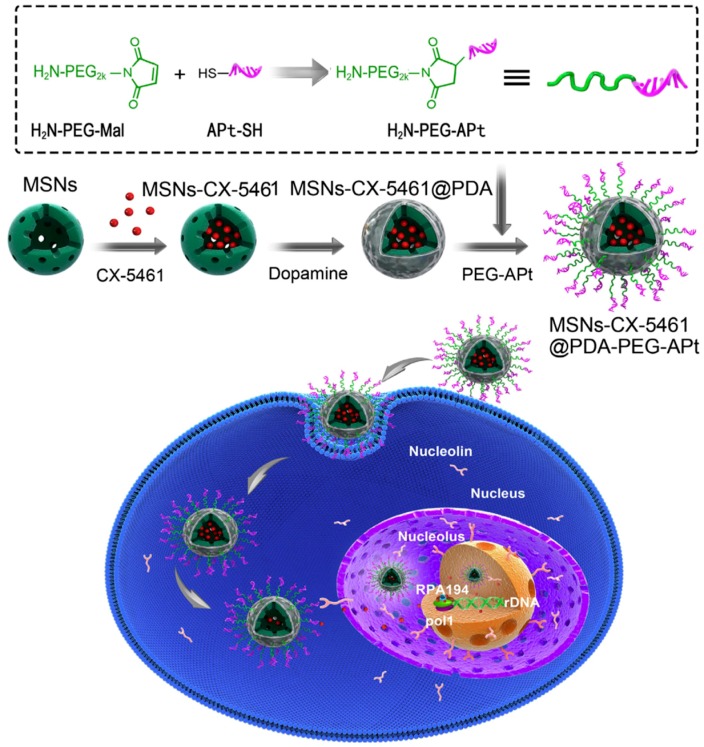
Aptamer-conjugated nanoparticles (NPs). Scheme of CX-5461-loaded mesoporous silica NPs modified with AS-1411 aptamers to increase nucleoli targeting. Reproduced with permission from Reference [34].

Aptamers are a class of short nucleic acid (DNA or RNA) comprising several nucleotides. Aptamers are small, highly sensitive, biodegradable, and have immunogenicity, making them good candidates for active targeting ligands [33]. In 2018, Duo et al. used the AS-1411 aptamer to target mesoporous silica NPs containing CX-5461 to the nucleus of tumor cells (Figure 3) [34]. The AS-1411 G-rich DNA aptamer specifically recognizes nucleolin, a protein upregulated in many cancer cell lines. Nucleolin, which is present in nucleoli, nucleoplasm, cytoplasm, and on cell surfaces, can facilitate transport of bound NPs to the nucleus after cellular uptake. CX-5461 is a well-known small-molecule inhibitor of rRNA synthesis that triggers prodeath autophagy in tumor cells. In the study by Duo et al., CX-5461-loaded MSNs were coated by polydopamine to increase loading stability, after which AS-1411 aptamers were conjugated on the surface of NPs. After treatment, the resulting NPs successfully accumulated in the nucleolus of HeLa cells and inhibited cell growth through induced prodeath autophagy. In a HeLa cell xenograft mice model, aptamer-modified NPs exhibited higher accumulation in tumors compared to NPs without aptamers. Correspondingly, tumor growth in vivo was effectively suppressed by the AS-1411 aptamer-modified NPs without significant toxicity.

Recently, Xi et al. used the GBI-10 aptamer for tumor-targeting of NPs [35]. GBI-10 is a single strand DNA aptamer that strongly interacts with tenascin-C, a protein overexpressed in the extracellular matrix (ECM) of pancreatic ductal adenocarcinoma. For enhanced tissue penetration and cellular uptake, the authors also simultaneously used a cell-penetrating peptide (CPP). The cell-penetrating function of the CPP is not specific to tumor cells, and it was shown that GBI-10 uses electrostatic attraction to avoid nonspecific accumulation at the site of injection. Dimeric camptothecin prodrug (CPTD) was loaded in these NPs modified with aptamer and CPP, which showed greater triggered release under high redox potential after cellular uptake. Cytotoxicity testing showed that the resulting GBI-10 aptamer and CPP-modified NPs containing CPTD (Apt/CPP-CPTD NPs) have a higher IC_50_ than CPP modified NPs (CPP-CPTD NPs) due to the camouflaged CPP. However, Xi et al. asserted that GBI-10 aptamers detach from Apt/CPP-CPTD NPs in tumors secondary to the high-affinity relationship between GBI-10 and tenascin-C. Recovered cell-penetrating ability of Apt/CPP-CPTD NPs was demonstrated by Miapaca 3D tumor spheroid microscopic images. Finally, the Apt/CPP-CPTD NPs were injected intravenously into orthotopic pancreatic cancer xenograft mouse models. Fluorescent images of mice also showed high accumulation in tumor sites at all time points. Accordingly, Apt/CPP-CPTD NPs showed enhanced antitumor efficacy and survival rates in vivo over other control groups, thereby demonstrating successful active targeting of NPs with the GBI-10 aptamer and CPP.

### 2.4. Small Molecules

Folate (FA) receptors are well known to be overexpressed in solid tumor cells and macrophages, making them attractive targets for many NPs via receptor-mediated endocytosis [36,37,38]. For example, Lv et al. prepared mesoporous silica NPs (MSNs) modified with FA for active targeting, and decorated NPs using the large gas-filled microbubble (MB) technique [39]. In this method, the gas-filled MB is destroyed under local ultrasound irradiation, resulting in the release of FA-modified MSNs across the endothelial layer and into the target tissue. Using this technique, one study loaded tanshinone IIA (TAN), a hydrophobic drug, into MSNs and demonstrated both a high loading capacity and potent ability to induce tumor cell apoptosis. The FA-modified MSNs and MB also showed negligible cytotoxicity in both HeLa and A549 cells without TAM and which expressed relatively high and low levels of the FA receptor, respectively. However, the MSN-FA-TAN-MB showed enhanced cellular uptake via receptor-mediated endocytosis and increased apoptosis of HeLa cells compared to A549 cells. During in vivo testing with an H22-tumor-bearing mouse model, intravenously injected MSN-FA-TAN-MB showed greater antitumor efficacy when the tumor site was irradiated with ultrasound. These results demonstrated that a combination strategy based on the ultrasound-guided releasing and FA-mediated active targeting of NPs could be used effectively for drug delivery.

Anisamide is a benzamide known to bind sigma-1 receptors overexpressed in cancer cells [40]. In 2017, Huo et al. showed improved vaccine therapy for melanoma using the tyrosinase-related protein 2 (Trp2) vaccine and sunitinib, a known tyrosine kinase inhibitor [41]. Sunitinib inhibits tyrosine kinase activity, thereby blocking tumor growth and inducing tumor apoptosis. As sigma receptors are overexpressed in melanoma, anisamide was employed as a targeting ligand to facilitate efficient delivery of sunitinib to melanoma tumors. Specifically, the authors prepared sunitinib base-loaded polymeric micelles (SUN_b-PM_) modified with anisamide. In B16F10-tumor bearing mice treated with these modified NPs, the tumor inhibition ratio was the greatest for Trp2 + SUN_b-PM_. Furthermore, as a result of the immune response elicited by Trp2, the number of CD8+ T cells in Trp2+SUN_b-PM_ groups was significantly increased. On the other hand, there was a decrease in the abundance of myeloid-derived suppressor cells and T regulator cells, both of which play important roles in immune suppression. They found that T helper 1 and 2 cytokine levels were appropriately altered in order to enhance antitumor immune responses. Based on these findings, the authors concluded that anisamide-modified NPs containing tyrosine kinase inhibitors combined with a vaccine may afford synergistic antitumor effects. The mechanism of anisamide remains controversial. The Leroux group insisted that cellular uptake of anisamide-modified NPs is not related to Sigma-1 receptors [42]. They also suggested the possibility that anisamide binds to Sigma-2 receptors instead of Sigma-1 receptors, and suggested that further studies are needed to identify the exact mechanism of action of anisamide [43].

**Figure 4:**
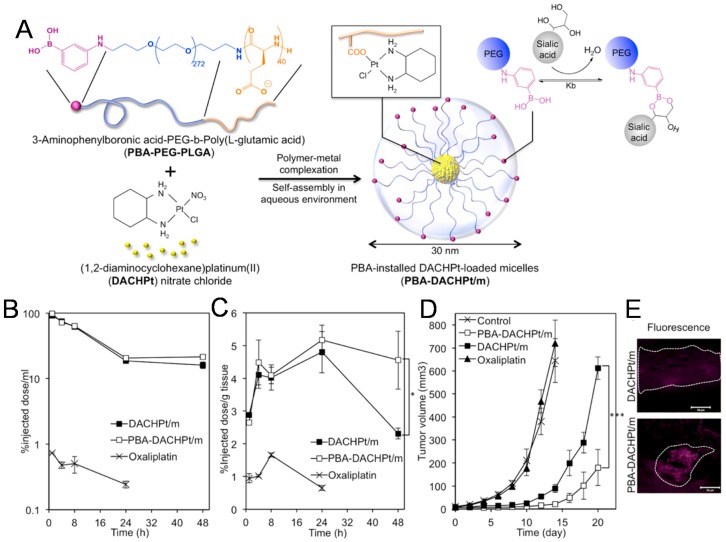
Small molecule-conjugated nanoparticles (NPs). (**A**) Scheme of phenylboronic acid (PBA)-conjugated dichloro-(1,2-diamino-cyclohexane)-platinum (II) (DACHPt)-loaded micelles (PBA-DACHPt/m) by self-assembly via polymer−metal complex formation. (**B**) Blood circulation time of intravenously injected DACHPt/m, PBA-DACHPt/m, and oxaliplatin. (**C**) Tumor accumulation of DACHPt/m and PBA-DACHPt/m in an orthotopic B16F10 mouse model. (**D**) Tumor volume of orthotopic B16F10 tumors after treatment with oxaliplatin (8 mg/kg), DACHPt/m, or PBA-DACHPt/m (3 mg/kg). (**E**) Ex vivo fluorescence images showing tumor tissue accumulation of DACHPt/m and PBA-DACHPt/m. Reproduced with permission from Reference [44].

In another example showing the feasibility of small molecule targeting ligands, the Kataoka group demonstrated that phenylboronic acid (PBA) strongly binds N-acetylneuraminic acids, which are the main components of sialic acid (SA) compared to other sugars including galactose, mannose, and glucose (Figure 4) [44]. In particular, the difference in binding affinity is further increased at pH 6.5, which is consistent with the acidic intratumoral environment. Taking into consideration the abundance of SA present on the surface of tumor cells, they designed phenylboronic acid (PBA)-installed micelles for drug delivery, specifically, the prepared PBA-modified poly-(ethylene glycol)-b-poly-(L-glutamic acid) (PEG-PLGA) micelles containing dichloro-(1,2-diamino-cyclohexane)-platinum (II) (DACHPt), an anticancer drug. After a nine-hour incubation in vitro, they found large amounts of internalization of PBA-modified micelles in B16F10 tumor cells, while control NPs without PBA exhibited decreased uptake. Further in vivo studies with B16F10 tumor-bearing mice showed that PBA-modified micelles containing DACHPt inhibited both tumor growth and metastasis, which was attributed to their excellent accumulation of tumor cells. However, PBA needs to be applied as a ligand for drug delivery carefully, because the amount of SA varies significantly among different cancer cell lines.

## 3. Conclusions

To date, we have summarized the representative examples of biological ligands for targeting of NPs. Various ligands including proteins, carbohydrates, nucleic acids, peptides, and small molecules are capable of increasing the specific binding of NPs containing drugs to disease cells to increase the efficacy of chemotherapy. Active targeting is a term that is often misunderstood as the ability of a ligand to control and direct the movement of conjugated NPs to target cells in vivo like guided missiles. However, dramatic changes in organ distribution of NPs do not occur in many cases, and the ligands present on NPs only help the binding of NPs on target cells. As observed in the studies of the Davis group, biodistribution and organ distribution of NPs in vivo changed only slightly following conjugation of biological ligands, especially on whole-body imaging [45]. Nevertheless, the ligands significantly enhanced the binding and uptake of NPs after reaching tumor tissue, which may improve therapeutic efficacies, not large-scale distribution [46].

Physicochemical properties such as size, shape, rigidity, or surface properties are very important to determine the large-scale distribution of NPs. It is known that nanoparticles from 10 to 500 nm can move through vessels and accumulate in tumor tissue [9]. Strong cationic charges increase liver accumulation and antifouling PEG modification helps tumor targeting [47]. Recent studies showed that soft NPs are advantageous for accumulation and penetration in tumor tissue [48,49]. In addition, the study of Reuter et al. showed unexpected lower tumor accumulation of RGD peptide-modified NPs compared to control PEG-modified NPs [50]. It demonstrated the importance of physicochemical properties of NPs and the unintended result of ligand modification. Thus, it is important for researchers to consider these properties while at the same time not overestimating the effect of biological ligands [46].

Another important consideration of NP ligands is their cost [51]. For example, antibodies are attractive ligands due to high specificity and diverse targets, but their production and conjugation cost a lot [52]. Development of more efficient methods for coupling of antibodies onto NPs may reduce the cost. From this point of view, small chemical molecules are generally cheaper than proteins or aptamers. Furthermore, first-generation nanomedicines including Doxil and Abraxane have insufficient specificity on their own, while many NPs using biological ligands remain in various stages of clinical development. We expect that drug-eluting, targeted NPs will become increasingly commercialized and available for use in the clinic in the near future to provide greater benefit to reduce side effects and improve therapeutic efficacy.

## Figures and Tables

**Table 1:** Ligands for active targeting of nanoparticle drug delivery systems.

Type	Ligands (Example)	Advantage/Disadvantage	References
Proteins	Antibodies, transferrin	High specificity/large size, low stability	[18,19,22]
Polysaccharides	Hyaluronic acid	Can be used as polymer backbone of nanoparticles/overexpressed receptors in liver tissue	[24]
Peptides	RGD, IL4RPep-1	Easy fabrication, small size/cleavable by peptidase	[26,29,31]
Aptamers	AS-1411, GBI-10	High specificity, small size/cleavable by nuclease, high cost	[34,35]
Small molecules	Folate, anisamide phenylboronic acid	Small size, very low cost/targets are also expressed in normal tissues	[39,41,44]
